# Patent Spring Washer Axles

**Published:** 1878-12

**Authors:** 


### Patent Spring Washer Axles.

Physicians of Buffalo have for a long time regarded the continuous jolting of our carriages over stone pavements as one of the causes of the *paralysis* so common in the older members of the profession. As to the truth of this proposition we have nothing now to say, but we learn that a new axle for our carriages is offered which promotes comfort, and is worthy of trial. Prof. E. M.

Moore, M. D., of Rochester, writes the following to the manufacturers, Messrs. Sheldon & Co., Auburn, N. Y. Full illustration in our advertising page:

MESSRS. SHELDON & Co.: *Dear Sirs*—I have used your axles but a short time and can have no knowledge of their durability, but am convinced of their possessing this quality from their structure. But I am very certain that they produce ease and comfert in riding. I have driven my buggy over rough places, and have been surprised at the improvement over its former condition. I feel that they have especially disposed of my pet horror—that is, crossing the rail of the street railroad obliquely. I have driven at a round trot across this renowned axle-breaker, at an angle of forty-five degrees, with but little jar. When driving on a Medina stone pavement the wheels seem to be in a state of vibration, but are ever kept true by the Spiral Springs.

Yours truly, E. M. MOORE.

